# Glioma-Associated Microglia Characterization in the Glioblastoma Microenvironment through a ‘Seed-and Soil’ Approach: A Systematic Review

**DOI:** 10.3390/brainsci12060718

**Published:** 2022-05-31

**Authors:** Grazia Menna, Pier Paolo Mattogno, Carlo Maria Donzelli, Lucia Lisi, Alessandro Olivi, Giuseppe Maria Della Pepa

**Affiliations:** 1 Institute of Neurosurgery, Fondazione Policlinico Universitario Agostino Gemelli IRCCS, 00168 Rome, Italy; mennagrazia@gmail.com (G.M.); pierpaolo.mattogno@policlinicogemelli.it (P.P.M.); carlomaria.donzelli01@icatt.it (C.M.D.); alessandro.olivi@policlinicogemelli.it (A.O.); 2 Institute of Pharmacology, Catholic University of Rome, 00168 Rome, Italy; lucia.lisi@unicatt.it

**Keywords:** glioblastoma, tumor microenvironment, glioma-associated microglia, immunological homeostasis, PD-L1

## Abstract

**Background and aim**: Ever since the discovery of tumor-associated immune cells, there has been growing interest in the understanding of the mechanisms underlying the crosstalk between these cells and tumor cells. A “seed and soil” approach has been recently introduced to describe the glioblastoma (GBM) landscape: tumor microenvironments act as fertile “soil” and interact with the “seed” (glial and stem cells compartment). In the following article, we provide a systematic review of the current evidence pertaining to the characterization of glioma-associated macrophages and microglia (GAMs) and microglia and macrophage cells in the glioma tumor microenvironment (TME). **Methods:** An online literature search was launched on PubMed Medline and Scopus using the following research string: “((Glioma associated macrophages OR GAM OR Microglia) AND (glioblastoma tumor microenvironment OR TME))”. The last search for articles pertinent to the topic was conducted in February 2022. **Results:** The search of the literature yielded a total of 349 results. A total of 235 studies were found to be relevant to our research question and were assessed for eligibility. Upon a full-text review, 58 articles were included in the review. The reviewed papers were further divided into three categories based on their focus: (1) Microglia maintenance of immunological homeostasis and protection against autoimmunity; (2) Microglia crosstalk with dedifferentiated and stem-like glioblastoma cells; (3) Microglia migratory behavior and its activation pattern. **Conclusions:** Aggressive growth, inevitable recurrence, and scarce response to immunotherapies are driving the necessity to focus on the GBM TME from a different perspective to possibly disentangle its role as a fertile ‘soil’ for tumor progression and identify within it feasible therapeutic targets. Against this background, our systematic review confirmed microglia to play a paramount role in promoting GBM progression and relapse after treatments. The correct and extensive understanding of microglia–glioma crosstalk could help in understanding the physiopathology of this complex disease, possibly opening scenarios for improvement of treatments.

## 1. Introduction

Ever since the discovery of tumor-associated immune cells, there has been growing interest in the understanding of the mechanisms underlying the crosstalk between these cells and tumor cells, which can range from conferring a growth advantage to the latter to enabling the cancer cells to escape autoimmunity. To achieve this, a significant effort has been made to define the types and characteristics of the cells constituting the tumor microenvironment (TME) and to characterize how these cells assist the tumor cells by producing cytokines, chemokines, growth factors, and triggering the release of inhibitory immune checkpoint proteins from T cells. 

Gliomas, with emphasis on glioblastomas (GBMs), are no exception and they present a wide range of glioma-associated macrophage and microglia (GAMs). Extensive literature in this field has been published to gain a better understanding of the composition of the TME and of the role that resident microglia and immune cells from peripheral blood play in ensuring an immunosuppressive environment in which the tumor can thrive undisturbed. 

The nature of the TME shapes both therapeutic responses and resistance, justifying the recent impetus to target its components. Despite the growing body of evidence on the topic, many aspects are yet to be explored in the field. Open questions are related to microglia distribution in the peritumoral area, its spatial and molecular interaction with GBM infiltrative margins and stem cell compartment, and the real extension of the so-called TME with respect to the tumoral core. A correct and extensive understanding of microglia–glioma crosstalk could help in understanding the physiopathology of this complex disease, possibly opening scenarios for improvement of treatments. An immunosuppressive pressure acts on the microglia and macrophage towards a polarization to an M2 protumor-immunosuppressive cellular phenotype. However, it is not ultimately clear to what extent an M2 macrophage distribution is capable of maintaining an immunologically downregulated environment.

A “seed and soil” approach has been recently introduced to describe the GBM landscape: the tumor microenvironment acts as fertile “soil” interacting with the “seed” (glial and stem cells compartment). Microenvironmental contribution seems critical to gaining a better understanding of the unique challenges GBM poses and could be pivotal in:

(a) developing new treatments targeting GAMs, thus rendering the tumor once again targetable by host immunity, and slowing its progression and aggressiveness by inhibiting the microglia and tumor cell crosstalk
(b) defining the hystotypes of the tumor and degree of response to treatment based on the GAMs composition and infiltration pattern.

In the following article, we provide a systematic review of the current evidence pertaining to the characterization of GAMs and microglia, and macrophage cells in the glioma TME.

## 2. Methods

This study was conducted in accordance with the PRISMA-P (Preferred Reporting Items for Systematic Review and Meta-Analysis Protocols) guidelines. An online literature search was launched on PubMed Medline and Scopus using the following research string: “((Glioma associated macrophages OR GAM OR Microglia) AND (glioblastoma tumor microenvironment OR TME))”. The last search for articles pertinent to the topic was conducted in February 2022. Two authors, C.M.D. and G.M., independently conducted the abstract screening for eligibility. Any discordance was solved by consensus with a third, senior author, G.D.P. No restrictions on the date of publication were made. Exclusion criteria were as follows: no comparative study design, studies published in languages other than English, and metaanalysis. A systematic abstract screening of the references (forward search) was performed to identify additional records.

## 3. Results

**Figure 1:**
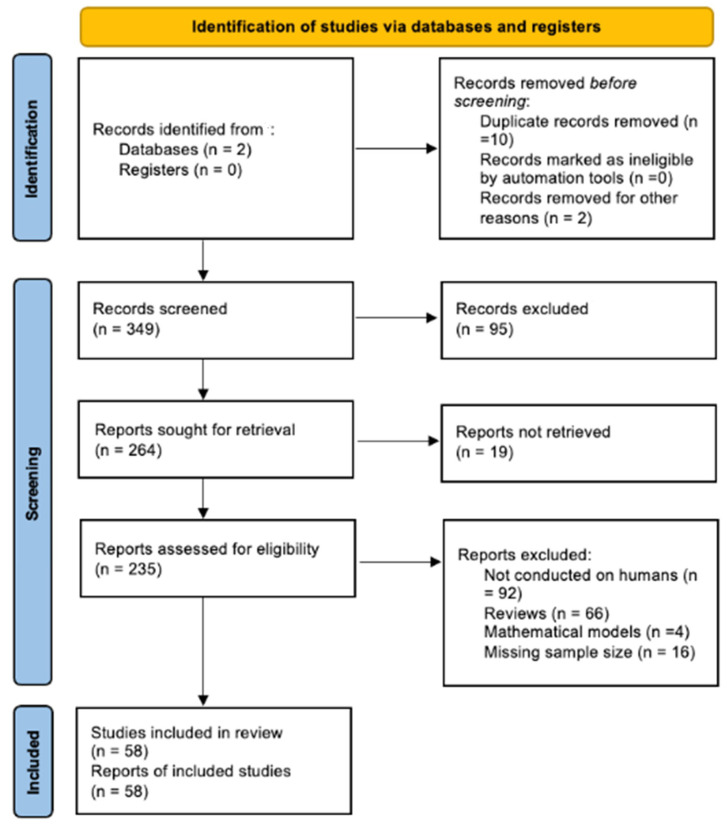
Prisma diagram showing the research strategy and selection of papers included.

The search of the literature yielded a total of 349 results. Duplicate records were then removed (*n* = 10), via title and abstract screening; 235 studies were found to be relevant to our research question and were assessed for eligibility (Figure 1). Upon a full-text review, 58 articles were included in the review. 

The reviewed papers were further divided into four categories based on their focus:

(1) Microglia maintenance of immunological homeostasis and protection against autoimmunity (Table 1);
(2) Microglia crosstalk with dedifferentiated and stem-like glioblastoma cells (Table 2);
(3) Microglia migratory behavior and its activation pattern (Table 3).

## 4. Discussion

**Figure 2:**
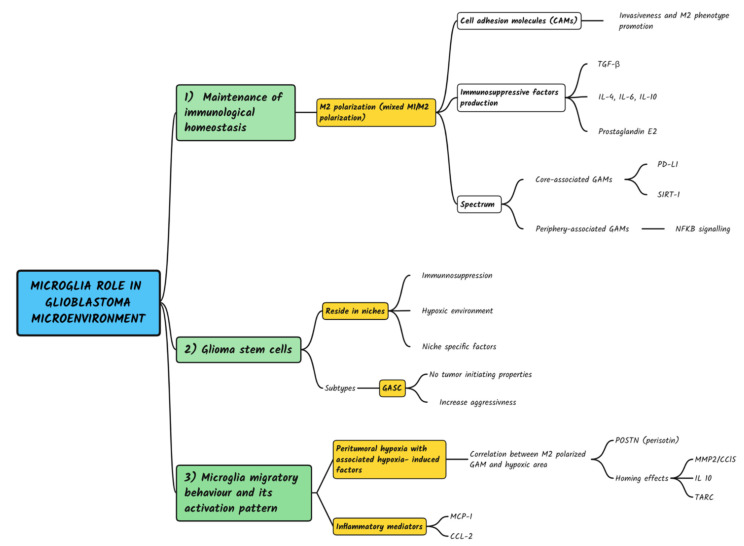
Schematic representation of multiple roles of microglia in Glioblastoma development, pathogenesis and relapse according a ‘seed and soil’ aproach.

GBM is a complex solid tumor with a highly inflammatory tumor environment. Approximately 30–50% of the brain tumor mass is constituted by microglia, monocytes, and macrophages, defined as GAMs [55]. These cellular populations are the major cerebral immune components [17], responsible for maintaining brain homeostasis, producing cytokines, chemokines, and growth factors (which altogether constitute the TME), and regulating tumor progression [56] (Figure 2).

### 4.1. Microglia Maintenance of Immunological Homeostasis and Protection against Autoimmunity

GBM immune-escape is primarily due to the characteristics of neoplastic cells [1,2,3,5,6,7], which mask tumor antigens with decreased expression of HLA molecules [8,9,10,11,12,13,14,15] and have the ability to produce immunosuppressive factors [57] and proapoptotic signals aimed at infiltrating tumor-specific immune cells [58,59]. In particular, the produced transforming growth factor-β (TGF-β) inhibits T-cell activation, proliferation, and differentiation, while promoting regulatory T-cells (T-regs) and suppressing both NK-cells and cytotoxic T-cells [55]. Complementarily, locally produced IL-10 has an inhibitory effect on T-helper (Th) cells, monocytes, macrophages, and dendritic cells (DC), resulting in an immune tolerance condition that favors tumor growth [9]. 

All the relevant findings regarding microglia maintenance of immunological homeostasis and protections against autoimmunity are summarized in Table 1.

GAMs constitute a crucial component of the TME, together with extracellular matrix (ECM) and other nonmalignant cells, such as endothelial cells, fibroblasts, and T lymphocytes which play a pivotal role in the development, growth, and malignant characteristics of gliomas [60]. Within this complex cellular multiplicity, different GAM components can be identified [2]: a first group originating from the embryonic yolk sac and considered central nervous system (CNS) immune tissue residents, and a second group consisting of the CNS-infiltrating leucocytes (e.g., monocytes, T, B, and natural killer cells), normally circulating within the blood vessels except in the case of a blood-brain barrier disruption, as described in inflammatory and neoplastic CNS pathologies [5]. Although monocytes, macrophages, and microglia have several common markers, such as an ionized calcium-binding adapter molecule 1 (Iba1) and CX3CR1, some studies regarding transcriptome analyses comparing microglia and other myeloid immune cells, have identified a multitude of genes dependent on the TGF-β signaling, codifying for Sall1, TGF-βr1, P2ry12, Fcrls, and Gpr34 that can be considered specific markers for microglia [61]. GAMs can exhibit a different spectrum of phenotypes. As generally accepted, the activated microglia and macrophages can present an antitumoral immune phenotype, defined as M1, through the secretion of proinflammatory cytokines, such as tumor necrosis factor-alpha, IL-1beta, and inducible nitric oxide synthase [1]. Alternatively, GAMs have also been reported to play a crucial role in some GBM features, such as growth, invasion, proliferation, and immunosuppression [62,63]. Particularly, GAMs are forced to switch to the M2 phenotypes in the GBM microenvironment, secreting factors such as IL-10, IL-4, IL-6, macrophage colony-stimulating factor, TGF-β, macrophage inhibitory factor, and prostaglandin E2, which, due to their anti-inflammatory action, facilitate tumoral immune-escape and increase tumor invasiveness, angiogenesis and growth, contributing to the creation of an immunosuppressive tumoral microenvironment [38]. However, in recent years different studies reported that M1 and M2 phenotypes would represent only extremes of broader and more articulated cell heterogeneity [64,65]. To reinforce this concept, Landry et al. report significant differences in the GAM populations present in the GBM core, compared to those of the GBM periphery: on one side, core GAMs manifest mainly a proinflammatory phenotype correlated with Programmed cell Death-1 (PD-1) signaling; on the other, peripheral GAMs exhibit an anti-inflammatory phenotype and a strong association with NFkB signaling [52].

Among the mechanisms that seem to support the proinflammatory activity of the GBM core, deacetylase Sirtuin 1 (SIRT1), whose gene is deleted in 80% of GBM tumors, seems to play a role. As a result, GBM cells continuously produce cytokines and factors attracting and activating glioma-associated microglia and macrophages, promoting a proinflammatory loop [7]. Another potential role in modulation and interaction between GBM cells and GAMs might be played by the ERp57/PDIA3 (protein disulfide-isomerase A3), an endoplasmic reticulum protein present both in GAMs and GBM cells, whose expression and activity were found to be directly proportional to the polarization capacity towards the protumor M2 phenotype of microglia [35]. This complex tumoral microenvironment, which mainly expresses immunosuppressive characteristics, especially in the tumoral periphery, determines a limited migration of T lymphocytes which represent less than 2% of the neoplastic cell mass [16]. This aspect has a pivotal role in therapeutic strategies, being immunotherapies targeting T cells, such as monoclonal antibodies against programmed cell death 1 (PD-1) or cytotoxic T-lymphocyte-associated protein 4 (CTLA-4), which are not suitable for treating GBM [66].

A further significant role in tumor metastasis and invasiveness is played by cell adhesion molecules (CAMs), which are cell surface-proteins that mediate cell–cell adhesion, particularly between immune cells and target tissues. Intercellular CAM (ICAM-1, also known as CD54) and vascular CAM (VCAM-1, also known as CD106), are upregulated by several proinflammatory cytokines, improving the immune-mediated response. Furthermore, ICAM-1 and VCAM-1 play important roles in the adhesion of cancer cells to the endothelium, especially in the context of the inflammatory microenvironment, supported by the high concentration of IL-1b, correlated with higher grade gliomas [57,67]. Through binding to IL-1R present on the surface of GBM cells, IL-1 can promote the cascade of the MAPK and p65 signaling pathways, resulting in the production of proteins and soluble forms of VCAM-1 and ICAM-1, which enhance monocytes adhesion and secretion of CCL2 and IL-6 through activated macrophages. This would seem to modulate the immunosuppressive activity, increasing the survival of myeloid monocytes recruited to the TME and polarizing their differentiation toward M2-type macrophages. Furthermore, the immunosuppressive microenvironment is not exclusive to GBMs: it has also been demonstrated in IDH mutant anaplastic astrocytomas and in IDH mutant/1p-19q codeleted anaplastic oligodendrogliomas [13].

### 4.2. Microglia Crosstalk with Dedifferentiated and Stem-like Glioblastoma Cells

Glioma stem cells (GCS) are a chemo-resistant population that can drive tumor growth and relapse. The traditional theory of cancer stem cells defines them as a minor subpopulation of self-renewing malignant cells that maintain a low but steady level of unlimited proliferation [57,68]. The latter maintains the tumor, and these cells’ low mitotic activity protects them from treatment approaches that are directed against actively dividing cells [29,69]. Therefore, these cells can survive treatment and give rise to recurrences [18,19,20,21,22,23,24]. In addition, stem cells interact with the TME, and their interplay is mandatory to develop biological resistance and sustain the tumorigenic process [31,39]. General features of GSCs are treatment resistance and association with tumor recurrence. They reside within specific anatomic niches, which can be seen as specialized microenvironments ensuring their stemness, proliferation, and apoptosis resistance, analogous to tissue stem cell niches. These niches shield GSC functionally by providing prosurvival cues and anatomically by blocking them from therapy exposure [18,30,33,41].

All the relevant findings regarding microglia crosstalk with dedifferentiated and stem-like glioblastoma cells are summarized in Table 2.

Four different niches have been identified, each one with a distinct TME composition and with niche-specific transcriptional and epigenetic signature. These are subarachnoid, perineuronal, perivascular, and perinecrotic [25,27,31,42,64,70,71]. Within these niches, GSCs are regulated by several mechanisms other than from the TME, such as [32,37,48,72]:

- Host immune system: since immunosuppression is a cardinal feature of malignant tumors.
- Metabolism: GBM is characterized by a hypoxic environment, which in turn increases the need for glycolysis, active in malignant cells, even under aerobic conditions. Hypoxic niches and perivascular niches have been reported both inside and outside of the tumor mass.
- Niche-specific factors: perivascular niches develop along capillaries and arterioles where GASCs are in direct contact with the endothelium. Invasive niches are characterized by perivascular growth of single invasive neoplastic cells along the capillaries, between the endothelium and reactive astrocytes.

Tumor-associated microglia and macrophages accumulate in perivascular and perinecrotic hypoxic niches, where they start a crosstalk with the staminal compartment ultimately promoting disease progression and relapse after treatments [17,37,39,40,41,73]. Mechanisms of crosstalk have only been investigated in a few seminal studies [29,32,33,34,35,36,51].

Both clinical outcomes and mathematical modeling show that GSCs are a key mechanism in determining the resistance of the whole tumor to therapy. Isolated cell lines of glioblastoma do not show such a marked resistance as observed in GBM patients. In addition to this, in the brain-mimetic biomaterial platform for the 3D culturing of patient-derived GBM cells, the modulation of hyaluronic acid content and mechanical properties of biomaterials were required to recreate the known resistance to epidermal growth factors receptor (EGFR). 

As previously discussed, TME niches play a multifaceted role in regulating GSCs and this motivates further investigation. Additionally, it has been demonstrated that GSCs immune evasion is critical to sustaining the tumorigenic process. GSCs express low levels of molecules involved in the processing and presenting tumor antigens to TCRs, a crucial stimulatory signal to the T-cell response. Consequently, they escape from recognition by antitumor immunity and possibly actively suppress T-cell activation. GSCs express various molecules that deliver either stimulatory or inhibitory signals during direct physical contact with tumor-infiltrating lymphocytes (TILs). The balance of these opposing signals regulates the amplitude and quality of TIL response and the aberrant activation of the inhibitory signals, also known as immune checkpoints, is a mechanism utilized by cancer cells to evade immune attacks [43,68].

Given the therapeutic potential and the promising therapies targeting the PD-1/PDL1 axis in GBM, the association between GSCs and the PDL1 axis in GBM deserves further analysis and investigation.

Hsu et al. recently demonstrated that epithelial-mesenchymal transition (EMT) enriches PD-L1 in GSCs by the EMT/β-catenin/STT3/PD-L1 signaling axis, in which EMT transcriptionally induces Nglycosyltransferase STT3 through β-catenin, and subsequent STT3-dependent PD-L1 Nglycosylation stabilizes and upregulates PD-L1. The axis is also utilized by the general cancer cell population, but it has a much more profound effect on GASCs as EMT induces more STT3 in CSCs than in nonGASCs. They further identified a noncanonical mesenchymal–epithelial transition (MET) activity of etoposide, which suppresses the EMT/β-catenin/STT3/PD-L1, leading to PD-L1 downregulation and sensitization of cancer cells to anti-Tim-3 therapy. On the one hand, this gives hope, on the other hand, it must be said that the expression of PD-L1 in GASC has never been described and further studies would be necessary to understand the possible potential for research and therapeutics [74].

This confirms GSCs and their interplay with TMEs to be mandatory for biological resistance.

Against this background, our group previously isolated a subpopulation of stem cells, called glioma-associated stem cells (GASCs). Indeed, GASCs are devoid of tumor-initiating properties, but show stem cell properties and the ability to support, in vitro, the biological aggressiveness of tumor cells [75].

In the infiltrating front of the tumor, transcriptomic suggested the presence of a GASC population possibly responsible for tumor recurrence. As already mentioned, even when GBM resection is performed beyond the tumor edge, there is no assurance that all tumor cells can be located and resected: infiltrating tumor cells are enriched with GASC, which in turn interacts with the TME, thus promoting tumor growth; in turn, the TME, by favoring hypoxic conditions, contributes to GASC generation.

### 4.3. Microglia Migratory Behavior and Its Activation Pattern

Despite a growing body of evidence, many aspects are yet to be explored regarding the migratory behavior of microglia with respect to tumoral margins. Open questions are related to microglia distribution in the peritumoral area, its spatial and molecular interaction with glioma infiltrative margins, and stem cell compartment [76]. Seminal studies showed how microglia move in a rather random way, whereas glioma cells exhibit a “committed” migratory behavior with significantly increased directionality compared to microglia [77]. However, it is unclear if glioma cells and microglia are responding to different migratory cues or are responding to the same cues but in different ways. GAMs polarization is influenced by both macrophage localization and tumor microenvironment signaling, resulting in a more complex scenario than the simple M1 and M2 activation status. Macrophage polarization in GBM has not yet been fully elucidated, and most results have been obtained in experimental nonhuman settings [46,47,48,49,50,78].

All the relevant findings regarding microglia migratory behavior and its activation pattern are summarized in Table 3.

Glioma cell–GAM crosstalking is fundamental to understanding microglia migratory behavior and its activation pattern in the center of the tumor and the surrounding periphery. Several studies have confirmed the central role of this crosstalk, highlighting a strong correlation between the local density of glioma cells and the rapidity of GAM migration and polarization [77,78]. Glioma cells stimulate the motility of microglial cells at the peritumoral infiltrative margins and after activation in a short period, microglia may enable more contact with cells via this random migration, resembling a surveillance function. However, microglia accumulation and polarization should not be regarded as a mere nonspecific reaction to tissue injury with consequent cytokine gradients, as it ultimately reflects their active participation in supporting and promoting the invasive phenotype of astrocytoma cells [44,51,52,53,79].

Recent evidence highlights that the migration of tumoral and microglia compartments is ‘driven’ by, (a) a condition of peritumoral hypoxia with associated hypoxia-induced factors and, (b) inflammatory mediators produced by immune cells in the TME. 

Hypoxia probably is the strongest determinant in shaping glioma and GAM migration. Indeed, it is well known that gliomas contain large hypoxic areas, and that a correlation between the density of M2-polarized GAMs and hypoxic areas exists. This suggests hypoxia plays a supportive role during GAM recruitment and M2 induction. Recent literature demonstrated perivascular niches in hypoxic areas and that hypoxia can affect chemotactic factors expression in such niches [46,49,80,81]. This mechanism underlies hypoxia-induced GAM recruitment and polarization, even if a clear description of M1- and M2-polarized cells distribution in a “topographic fashion” is still lacking in literature [47].

To date, in normal brains, the macrophage population is mainly located in perivascular (Virchow–Robin) spaces of cerebral microvessels. These are continuously repopulated by blood-derived monocytes and macrophages, and more rarely by resident brain microglia [47]. A considerable amount of M2 macrophages can be identified in perinecrotic and perivascular areas, which are indicators of an advanced stage of the tumor. The accumulation of GAMs in avascular and necrotic areas strongly accounts for their exposure to hypoxia, recognized as a key stimulus for alternative macrophage activation. Altogether, these data further support the notion that the macrophage phenotype could be a result of glioma progression owing to an interactive network involving glioma stem cells, proinflammatory-activated glioma cells, GAMs, and other components of the perivascular niche [76]. Recent studies confirmed a high prevalence of M2-polarized macrophages mainly disposed around the clusters of proliferating vessels. However, it must be acknowledged that whereas ‘quantitative’ information on GAM has been quite widely investigated, little is known about GAM spatial distribution within and around tumor mass and if a possible ‘gradient of activation status’ at the tumor periphery exists. 

Furthermore, Guo et al. showed how hypoxia-increased periostin (POSTN) expression in glioma cells actively promotes the recruitment of macrophages and that hypoxia-inducible POSTN expression was increased by TGF-α via the RTK/PI3K pathway [46].

Other studies have shed light on the mechanisms that underlie GAM recruitment and M2 polarization under hypoxic conditions in gliomas. Specific chemokine or metalloproteinase upregulation has shown a distinct ‘homing effect’ with regards to GAMs. As shown in the seminal study by Yu-Ju et al., chemokine C-C ligand 5 (CCL5) modulates the migratory and invasive activities of glioma cells in association with metalloproteinase 2 (MMP2) expression. In response to CCL5, glioma cells undergo a synchronized increase in intracellular calcium levels and glioma cells tend to migrate toward GAM-conditioned media activated by a granulocyte-macrophage colony-stimulating factor (GM-CSF) in which CCL5 is abundant. Moreover, an association between CCL5 and GAM activation has been demonstrated [51]. Authors suggest that modulation of glioma calcium levels may restrict the effect of CCL5 on glioma invasion and could be a potential therapeutic target for alleviating glioma growth. Indeed, GBM expresses a plethora of macrophage chemoattractants, such as IL-10, macrophage migration inhibitory factor (MIF), cytokines of the CSF family (macrophage colony-stimulating factor1, M-CSF, and granulocyte-macrophage colony-stimulating factor, GM-CSF), monocyte chemotactic protein 1 (MCP-1), alternative macrophage activation-associated CC chemokine-1 (AMAC-1), thymus and activation regulated chemokine (TARC) belonging to the CC chemokine family, chemokine (C-X-C motif) ligand 4 (CXCL4), chemokine (C-X3-C motif) ligand 1 (CX3CL1/fractalkine), and stromal cell-derived factor 1 (SDF-1). 

Regarding inflammatory mediators, it has been explained in the previous sections how inflammatory cytokines are determinants in GAM polarization and activation [2]. CC motif chemokine ligand 2 (CCL-2), also known as monocyte chemoattractant protein 1 (MCP-1), is a major chemokine that acts as a glioma cell-derived monocyte chemotactic factor. Indeed, overexpression of CCL-2 results in increased migration. Conversely, stromal cells such as macrophages secrete more CCL-2 into the TME than cancer cells. In a positive feedback loop, GBM cells secrete CCL-2 and attract macrophages and microglia; these cells also secrete CCL-2, and the number of activated cells is increased. Thus, GBM cells, macrophages, and microglia exhibit a robust reciprocal network in proliferation and migration. Other authors showed how chemokine receptor pairs CXCL12/CXCR4/CXCR7, CXCL16/CXCR6, and CX3CL1/CX3CR1 are involved in tumor progression and that GBM-associated macrophages and microglia are also characterized by expression of these chemokine receptor pairs indicating a pivotal role of this expression profile in GAM biology in gliomas [44,82].

### 4.4. Targeting the Tumor Microenvironment: The Disillusionment with Current Immunotherapeutic Treatment

Targeting therapeutics to the TME offers promise to improve patient survival and quality of life. Unfortunately, most of the clinically tested GBM-targeted therapies have shown little efficacy so far, such as erlotinib targeting the often overexpressed EGFR. At present, the anti-VEGF bevacizumab is the only drug targeting GBM TMEs that is approved by the US Food and Drug Administration (FDA). In addition, traditional treatments, radiotherapy, and chemotherapy with TMZ have shown to promote TME remodeling [83,84,85,86,87]:

- Radiotherapy improves the blood-brain barrier (BBB) permeability to chemotherapy; triggers TME remodeling via increased GAM infiltrates and improved GSC radiation resistance by activating DNA damage checkpoints to repair DNA damage.
- Temozolomide (TMZ) triggers a proinvasive TME phenotype by altering proteoglycans and glycosaminoglycans (GAGs) content.

These observations confirm that the TME acts as a “plastic soil” and can change its functions based on environmental stimuli [28,87,88,89,90,91,92,93,94].

As already stressed, TME niches play a multifaceted role in regulating GSCs and GSC immune evasion is critical to sustaining the tumorigenic process. GSCs express low levels of molecules involved in the processing and presenting of tumor antigens to TCRs, a crucial stimulatory signal to the T-cell response. Consequently, they escape from recognition by antitumor immunity and possibly actively suppress T-cell activation. GSCs express various molecules that deliver either stimulatory or inhibitory signals during direct physical contact with TILs. The balance of these opposing signals regulates the amplitude and quality of TIL response, and aberrant activation of the inhibitory signals, also known as immune checkpoints, is a mechanism utilized by cancer cells to evade immune attacks.

PD-1 belongs to the family of immunoglobulins and is expressed predominantly by activated T lymphocytes. It is often activated by PD-L1, one of the ligands known to be expressed by antigen-presenting cells (APCs), B lymphocytes, and parenchymal cells. Importantly, the expression of PD-L1 has been detected in glioma. In normal conditions, PD-1/PD-L1 engagement occurs controlling a prolonged activation of the immune system, often avoiding autoimmunity processes. Therefore, the PD-1/PD-L1 pathway has been appropriated by tumor cells to resist antitumor responses and facilitate tumor survival. Early immunotherapeutic attempts were focused on targeting PD-1 expression in the general cancer cell population. However, failure of anti-PD1 therapy has been seen in Checkmate 143, 498, and 548 clinical trials [95,96,97].

Thus, the focus has been shifted to PD-L1. In the TME, PD-L1 is regulated mainly by cytokine, while receptor antigen signaling is influenced by hypoxia, cytokines, and oncogenes. GBM cells express PD-L1, which engages with the PD-1 receptor primarily on T cells and attenuates its functions, effectively reducing the antitumor activity of these cells. Nevertheless, studies have shown heterogeneity of PD-L1 expression in tumor mass; a greater expression was observed at the edges of the tumor than in the core. Several phase I and II trials are focusing on PD-L1 in gliomas. Current evidence demonstrates that:

(1) PD-L1 quantitative expression has an impact on survival, independently of gender and age [98].
(2) PD-L1 overexpression is significantly associated with poor OS for patients from Asia and America, while no significant association for the survival of patients from Europe. This “ethnic bias” of PD-L1 has been observed in several clinical studies for patients with other solid tumors, such as KEYNOTE-161 in esophageal squamous cell carcinoma and KEYNOTE-063 in advanced gastric or gastro-esophageal junction cancer [98].
(3) IDH1-wildtype status in glioblastoma was PD-L1 expression positive, suggesting PD-L1/IDH1-wildtype association. From a molecular point of view, it could be that IDH1 mutation results in PD-L1 promoter hypermethylation, thus downregulating the expression of PD-L1. Therefore, PD-L1 immune checkpoint inhibitors analysis might not be advisable because of the globally low PD-L1 expression in patients with IDH1-mutant glioblastomas.

To sum up, higher expression of PD-L1 (both at protein and mRNA levels) is linked to a worse outcome. PD-L1 may expand and maintain immunosuppressive Tregs, which are associated with decreased survival in glioma patients. Beyond the direct impact on effector cells, the blockade of the PD-L1/PD-1 axis may reduce Treg expansion and further improve T cell function [98].

## 5. Conclusions

Microenvironmental contribution seems therefore critical to gaining a better understanding of the unique challenges GBM poses. Aggressive growth, inevitable recurrence, and scarce response to immunotherapies are driving the necessity to focus on GBM TMEs from a different perspective to possibly disentangle its role as a fertile ‘soil’ for tumor progression and identify in it feasible therapeutic targets. Against this background, our systematic review confirmed:

(1) Microglia play a paramount role in the maintenance of immunological homeostasis and protection against autoimmunity and its activation pattern at the TME level, polarized toward an M2 phenotype as selected by environmental pressure. This suggests that further investigation of microglia phenotypic characterization at the microenvironment level (M1 vs. M2 phenotype) is needed.
(2) Microglia crosstalk with dedifferentiated and stem-like glioblastoma cells in perivascular and perinecrotic hypoxic niches, where they start crosstalk with the staminal compartment ultimately promoting disease progression and relapse after treatments.
(3) Microglia demonstrate migratory behavior with respect to infiltrative margins of tumor cells. However, there are still many issues to be investigated. While the classification of macrophages or microglial cells into the M1 or M2 polarized state is a well-established approach in most preclinical models, the same is not true in the clinical research setting, because of the high degree of diversity and plasticity shown by these cell types. Therefore, dichotomizing GAMS into M1 and M2 activation status might be over simplistic as, indeed, a clear distinction between these phenotypes cannot be clearly distinguished. The resulting definitions of transcriptomic-based functional phenotypes of GAMs from human and experimental rodent gliomas are conflicting and indicate a mixture of M1 and M2 phenotypes. Cells within the tumor often display a complex pattern of phenotypes, upregulating both M1 and M2 molecular markers, and the prevalence of one phenotype on the other might also depend on the stage of disease [99].

In addition, the danger of ‘oversimplification’ goes along with the lack of universally recognized markers of the functional phenotype of GAMs. Although the association of GAM subtypes and patient overall survival has been observed in several papers still no consensus exists on reliable gene expression-based markers [100]. 

To further confound the research scenario on the topic it should also take into account that it is hard to define realistic research models as significant differences between human and mice models exists in terms of microglia polarization and inconsistency between rodent and human GAMs, regarding markers, has been reported. Despite some similarities, the mouse and rat models represent different pathways of GAM activation; comparative analysis of GAM transcriptomics across different in vivo models of human, mouse, and rat, failed to reproduce consistent microglia phenotypes that could be classified according to previously reported gene signatures and showed remarkably low similarity between models [99].

Despite the growing body of evidence on the topic reported in this paper, many aspects are yet to be explored in the field [4,26,45,54,98,101,102]. The correct and extensive understanding of microglia–glioma crosstalk could help in understanding the physiopathology of this complex disease, possibly opening scenarios for improvement of surgical strategies and medical treatments.

## Figures and Tables

**Table 1:** Microglia maintenance of immunological homeostasis and protection against autoimmunity.

Author	Year (dd-mm-yyyy)	Type of Study	Sample Size	Microglia Population	Sample	Outcome	Observations
Prosniak et al. [1]	5 June 2013	Genomics and immunohistochemistry	143	Monocytes	Primary tumor or epilepsy samples as control	Grade II and IV astrocytomas can be clearly differentiated based on the expression of certain M2 markers (CD14, CD68, CD163, and CD204, which appear to be elevated, MDSC and CD15, which instead are reduced) in tumor tissues, whereas grade III astrocytomas exhibit a range of expression between the lower and higher grade specimens. The content of CD163þ cells distinguishes grade III astrocytoma subsets with different prognoses.	Levels of mRNAs specific for IL-10, TGF-β1, and monocyte and M2 phenotypic markers were found to concordantly increase with WHO glioma grade.
Hatterman et al. [2]	23 May 2014	Proteomics/ immunofluorescence	20 humans	Macrophages	Tumor only	The mean expression of certain chemokines in GAMs (This population is characterized by CD11b+/CD45dim (microglia) and CD11b+/CD45high (macro- phages) phenotypes) is higher than in in vitro polarized M1 and M2 macrophages (M2-macrophages show elevated expression of the mannose (CD204/206) and scavenger receptor (CD163) and TGF-β1 and IL-10. M1-macrophages show L-1β, tumor necrosis factor (TNF)-α, interleukin (IL)-6 or IL-12 along with the ability to induce Th1-mediated immune responses) and displayed a very heterogeneous expression pattern with greater differences between single TAM-enriched fractions.	Comparison between TAMs and in vitro polarized M1- and M2-macrophages based on the expression of specific markers and chemokines.
Zeiner et al. [3]	29 August 2014	Cellular culture and histopathology	303	Macrophages	Tumor core, infiltration zone, and normal-appearing brain	This study provides evidence that CD74 expression is restricted to GAMs in gliomas contrasting findings of several other peripheral tumor entities.	As CD74 protein expression was absent from glioma cells but strongly present in glioma whole tissue, it was hypothesized that CD74 expression derives from non-neoplastic cells of the glioma microenvironment.
Meiczkowski et al. [4]	29 September 2015	Genomics	49	Macrophages	Not specified	The study uncovers new mechanisms by which defective ikkβ/nfκb signaling in myeloid cells affects antitumor immunity and facilitates glioblastoma progression.	Computational analysis of the immune response and TLR signaling genes led to the main finding of the reduced expression of IKBKB (a gene coding for ikkβ) in high-grade gliomas.
Zeiner et al. [5]	3 December 2018	Cellular culture and histopathology	344	Macrophages	Microarrays	This study provides evidence that the immune polarization phenotype of GAMs (Microglia/macrophage (M/M) are identified through CD68, CD163, CD206, and Iba-1. GAMs were isolated using CD11b and later characterized using Iba1, MCHII and CD45) might be distinct from TAMs in non-CNS cancers.	This study identified a rather mixed polarization phenotype with parallel expression of presumptive M1 and M2 markers and without evidence for an unequivocal M1 (*CCL2, CCL4, CCL3, IL1B, TLR2, CD86, CCL5* and *PTGS2)* or M2 (*MRC1, LGMN, IL10, MSR1, CD14, CD163)* polarization state of GAMs according to the M1 and M2 model.
Lin et al. [6]	28 June 2018	Genomics	Not specified	Macrophages	Tumor	In contrast to adult GBM, the immune microenvironment of DIPG is noninflammatory and does not contain a significant adaptive immune component.	The data presented here indicate that DIPG-associated macrophages are strikingly less inflammatory compared to adult GBM-associated macrophages.
Waters et al. [7]	29 May 2019	Cellular culture	Not specified	Macrophages and astrocytes	Not specified	Although relb coordinates anti-inflammatory feedback in astrocytes, this mechanism does not function in GBM cells due to both the limited activity of SIRT1 and the presence of YY1 in the nuclei.	These data strongly support the idea that p52-independent relb signaling is critical in GBM development or progression.
Martinez-Lage et al. [8]	29 November 2019	Immunohistochemistry	98	Monocytes and T cells	Tumor	GBMs demonstrate high levels of intratumor heterogeneity in immune infiltrate, GBMs subtypes vary significantly in the percentage of immune cells in their microenvironment, and mesenchymal GBMs have the highest percentage of microglia, macrophage, and lymphocyte infiltration.	Found trends across the GBM subtypes for most cell types, there was also significant variation within the subtypes. The results demonstrate that the mesenchymal subtype has the highest degree of immune infiltration for all the populations evaluated. Thus, mesenchymal GBM may be the most immunogenic.
González-tablas et al. [9]	3 December 2020	Flow cytometry	55	All immune cells	Tumor	This study shows that immune cell infiltrates, which consist of distinct immune profiles, are systematically present at highly variable levels in GBM.	Combined staining for GFAP and CD45, allowed clear-cut discrimination between GFAP^+^CD45^-^ GBM tumor and normal astrocytic cells and GFAP^-^CD45^+^immune cells.
Fu et al. [10]	4 February 2020	Mass cytometry	14	Monocytes and T cells	Tumor and peripheral blood	The immune cell composition of DA and OG was similar, and T cells in both diseases showed similar exhaustion characteristics. However, GAMs in DAs expressed higher levels of VEGF and TGF-β and exhibited more adverse immune-inhibitory characteristics than OGs.	The enrichment of exhausted T cell subpopulations, recruitment of Tregs, and the strong protumor phenotype of GAMs together contribute to the immunosuppressive microenvironment in DAs. GAMs of das exhibit more inhibitory characteristics than those of OGs.
Klemm et al. [11]	28 May 2020	Cellular culture, genomics, proteinomics, and more	Not specified	Macrophages	Not specified	By exploring the broad immune landscape, several pronounced differences between gliomas and BrMs were uncovered when directly compared side by side.	Gliomas contain an abundance of TAMs, whereas T cells were much fewer, particularly in IDH mut tumors. The clinical BrM samples showed a pronounced accumulation of lymphocytes and neutrophils.
Zinnhardt et al. [12]	12 February 2020	Imaging and histopathology	9	Myeloid cells	Tumor	A novel preoperative imaging protocol, including CE MRI, [18F]FET-, and [18F]DPA-714-PET-MRI, may allow comprehensive characterization of glioma extent and the heterogeneity of the immune tumor microenvironment in LGG and HGG.	The combination with the commonly used tracer [18F]FET provided complementary information about the glioma microenvironment, the degree of GAM infiltration and differential spatial extent, and the degree of tracer uptake.
Fu et al. [13]	4 February 2021	Mass cytometry	15	Macrophages	Tumor and peripheral blood	In this study, using cytof analyses, infiltrating immunocytes from surgically resected initial AG tissues, including aamut and AOD samples were analyzed.	It was verified that mononuclear phagocytes and T cells were the most abundant groups in the immune microenvironment of ags. Compared to that in the PBMCs, the ratios of immune checkpoint-positive-exhausted CD4+ T cells and CD8+ T cells were distinctly higher at the AG tumor sites
Cai et al. [14]	29 April 2021	Genomics	1395	Macrophages	Databases from TCGA and CCGA	The GASC is an important cell type in the microenvironment and might influence immunotherapy responses.	A strong correlation between the GASC score and tumorigenic cytokine score was found, indicating the tumor-supporting function of GASCs.
Y. Zhang et al. [15]	8 December 2021	Transcriptomics	1619 cases from three datasets	Macrophages	Not specified	An immune gene signature was constructed and showed a favorable efficiency in predicting the prognosis of patients with glioma. A high-risk score of this signature predicted enrichment of macrophages and less response to ICB therapy in glioma.	The higher complexity of immune cells was shown in high-risk patients, and the risk score was significantly positively correlated with the abundance of macrophages. Genes in our signature are highly expressed in macrophages by an open single-cell sequencing dataset of GBM. Patients with a high-risk score showed enrichment of macrophage in a tumor-supportive state, but not an antitumor state.
Cui et al. [16]	9 August 2021	Genomics	9 humans	TAMs	Not specified	The comprehensive characterization of immune cells from a total of nine GBM tissues revealed a unique immune landscape in GBM at the single-cell level and identified SPI1 as a potential immunotherapeutic target against TAMs in GBM.	The study’s results confirm that TAMs may occupy over one-third of the GBM tumor, with the ratio of the bone marrow-derived macrophages and the brain-resident microglia being 2:1, but infiltrating T lymphocytes comprising less than 2% of the tumor mass. SPI1 was a vital regulon in all states of TAMs
Shen et al. [17]	15 October 2021	Transcriptomics	3810	Monocytes and T cells	Datasets from NCBI, *ICGC,* CGGA	This reveals two molecular subtypes (i.e., C1/2) of brain tumors featured by distinct immune infiltration signatures and prognosis.	The C1/2 subtypes can distinguish glioma patients with different prognoses stratified by histology, tumor grade, and genomic alteration. In addition, the C1/2 subtypes can also reflect differences in microvascular infiltration, distant metastasis, and the radio-chemotherapy response of patients.

**Table 2:** Microglia crosstalk with dedifferentiated and stem-like glioblastoma.

Author	Year (dd-mm-yyyy)	Type of Study	Sample Size	Microglia Population	Sample	Outcome	Observations
Fries et al. [18]	10 January 1996	Cellular culture	26	Monocytes	Peripheral blood	Demonstrated that glioblastoma-associated peripheral blood monocytes release substantially larger amounts of EGF even over a long period (100 days) than monocytes from healthy individuals or patients with nontumorous diseases.	First study that demonstrated that glioblastoma-associated circulating monocytes release EGF. This study confirms findings that glioblastomas retain large numbers of EGFR-positive cells and large numbers of tumor-infiltrating monocytes as well.
Dziurzynski et al. [19]	13 April 2011	Cellular culture	Not specified	Macrophages	Not specified	The data argue against the idea that CMV merely plays a bystander role in glioblastoma pathology but is contributing to oncogenesis.	A tropism for CMV antigen expression was found, specifically pp65, in the GCSCs and MS microglia. Finding of production of CMV IL-10 by the GCSCs and its subsequent effect on the MS microglia precursor, the monocyte. CMV IL-10 activates IE1 in monocytes and there is conversion to the immunosuppressive phenotype M2.
J. Kmiecik et al. [20]	22 August 2013	Histopathological	65	T cells	Not specified	This study showed the beneficial role of immune cell infiltration into the tumor in GBM patients, despite multiple mechanisms of tumor immune escape.	Significant positive correlation of increased cd3+ and cd8+ cellular infiltration into the tumor with improved patient survival.
Shimato et al. [21]	9 October 2013	Cellular culture	13	Monocytes	Tumors and brains from healthy donors	These data indicate that GBM-mediated suppression of tumor-associated myeloid cell function is mediated at least in part by CAV1, and importantly, that activity can be restored by suppressing CAV1.	Upregulation of both AXL and Caveolin-1 (CAV1) has been demonstrated to reduce inflammation via inhibition of TNF- alpha production and was therefore investigated further. There was significant upregulation of CAV1 and AXL when monocytes were stimulated with LPS in the presence of GBMs compared to stimulated monocytes alone as measured by quantitative PCR.
Silver et al. [22]	25 September 2013	Cellular culture	4	Not specified	Tumor	The presence of heavily glycosylated, microenvironmental cspgs inversely correlates with the invasive character of human glioma.	Invasion of high-grade glioma occurs in the absence of a CS-GAG-rich inhibitory matrix. The absence of glycosylated cspgs provides favorable conditions for diffuse infiltration that typifies high-grade glioma.
Su et al. [23]	26 February 2015	Imaging and histopathology	22	GAMs	Tumor only	This study proved that TSPO imaging has the potential to detect early anaplastic transformation of lggs, and it can be effective to stratify patients with glioma who are suitable for TSPO-targeted treatment.	TSPO is predominantly expressed in neoplastic cells, with GAMs only partially contributing to PET signal and no expression in reactive astrocytes.
Choi et al. [24]	5 June 2015	Genomics	10	Macrophages	Not specified	The data suggest a putative neuroprotective role of TAMs by taking up excess extracellular glutamate by increasing expressions of glutamate transporters and glutamine synthetase.	This is an immunosuppressive, anti-inflammatory milieu that led to alterations in gene expression of glutamate receptors and transporters as well as GS (glutamine synthase) that were found to differ between TAMs and mdms.
J.P. Dijksterhuis et al. [25]	25 October 2015	Immunohistochemistry	48	Macrophages	Tumor core and eight cores of normal tissue	The results show that WNT-5A is the only re- presentative of the WNT family upregulated in human glioma compared to nonmalignant control brain tissue.	There is an approximately 4-fold increase in WNT-5A expression between nonmalignant control brain tissue and GBM patients’ brain tissue. The highest levels of WNT-5A were found in mesenchymal GBM.
M. D. Sørensen et al. [26]	2 August 2017	Histopathological	314 from different cohorts	Not specified	Tumor, tumor microarrays	Results demonstrate that the prognostic impact of TAMs in gliomas does not depend on the total amount of TAMs but on their acquired functional phenotype.	High levels of the M2-related marker CD204 correlated with increasing malignancy grade and poor patient survival in grades III and IV independently of clinical-pathological parameters. CD204+ TAMs were associated with a more aggressive tumor subtype and expressed proteins that could enable tumor progression.
Zhu et al. [27]	4 January 2017	Cellular culture	Not specified	Macrophages	From biobank	Demonstration of CECR1-mediated crosstalk mechanism between macrophages and glioma cells.	The influence of CECR1 on immune cells, and on macrophage polarization especially, seems to predominantly impact the systemic and cerebral vasculature and could affect the tumor vasculature in GBM. Expression analysis of typical M1 and m2a, b, and c subtype markers validated the presence of these TAM subtypes in human gliomas.
Wang et al. [28]	10 July 2017	Transcriptomics	53	Monocytes and T cells	Tumor	This study defines a strategy to determine transcriptional subtypes and associates expression subtypes to the tumor-associated immuno-environment.	The transcriptional glioma subtypes, defined through clustering based on tumor-intrinsic genes, strongly over lapped with the PN, CL, and MES subtypes, but identified the NE subtype as normal NE lineage contamination.
Li et al. [29]	26 October 2017	Cellular culture and histopathology	80	Microglia	Tumor and three healthy controls	Results indicate that SERPINA3 could play a critical role in glioma initiation and progression process.	Examination of the expression pattern of SERPINA3 at the various stages of glioma progression, including normal brain, pilocytic astrocytomas, diffuse astrocytomas, oligodendrocytes astrocytoma, anaplastic astrocytomas, and glioblastomas multiforme.
Caponegro et al. [30]	2 November 2018	Genomics	667 from TCGA	Macrophages	RNA-seq data	NRP1 expression is correlated with poor prognosis and glioma grade, and associates with the mesenchymal GB subtype. In human GB, NRP1 expression is highly correlated with markers of monocytes and macrophages, as well as genes that contribute to the protumorigenic phenotype of these cells.	Functional gene analysis suggests that NRP1 is associated with markers of monocytic infiltration and protumorigenic GAMs in human GB. The results demonstrate that both LGG and GB patients with high NRP1 expression have enriched monocytic, macrophage, and M2 macrophage populations. AIF1 and ITGAM (Iba1 and CD11b, respectively) are pan markers of monocytes, macrophages, and microglia, and are highly upregulated across human GB subtypes. Significantly correlated with genes that characterize the M2 pro-tumorigenic GAM signature, such as Adm and Mrc1. Also, significantly correlated with TMEM119 and TMEM173.
Sadahiro et al. [31].	12 March 2018	Cellular culture and xenograft	Not specified	Not specified	Patient-derived GBM (neuro)sphere cultures	This study identified that tumor-associated MG/Mø cells produce PROS1 that binds and activates AXL in GSCS. The PROS1/AXL signaling axis in GSCS subsequently activates the tumor-intrinsic nfkb pathway to promote the MES phenotype of GBM tumors.	Negative correlation between the expression of PDGFRA and PAXL in GSCS and GBM tumors. High AXL and PROS1 expression is associated with a poor prognosis in patients with GBM. The study demonstrates a role for PROS1-mediated AXL signaling in cancer, which is negatively regulated by pdgfra.
Couto et al. [32]	6 March 2019	Cellular culture	Not specified	GAMs	Cell lines from biobank	The data unravel a new oncogenic role for IL-6 in GBM through direct effects in microvascular ECs and regulation of endothelial permeability with a negative impact on brain barrier functions.	Data demonstrated significant alterations in the barrier properties of human ECs (endothelial cells) when exposed to cm-cc (conditioned medium of coculture), namely an increase in the permeability to smaller (4 kda) and larger (70 kda) molecular size dextrans and a decrease in the teer (transendothelial electric resistance) values.
Gjorgjevski et al. [33]	20 June 2019	Qpcr	20	Macrophages	Tumor	This study establishes M1- and M2-like markers CXCL10 and CCL13 for informative and reliable detection of GBM-associated microglia and macrophage polarization in conjunction with a defined protease profile as molecular determinants for GBM progression.	It was found that MMP9 and MMP14 are negatively correlated with GBM patient survival and associated with the markers to define a more M2-like microglia and macrophage phenotype.
L. Lisi et al. [34]	10 June 2019	Histopathological	42	Macrophages	Tumor core and tumor periphery	The mTOR pathway is activated in 39% of microglia–macrophage within GBM tumors, compared to 21% in peripheral tissues.	The mTOR pathway is fully activated in microglia cells under conditions mimicking the human GBM pathology. Microglia express an M2 protumor phenotype in the presence of glioma cells, and this M2 phenotype is downregulated in the presence of an mTOR inhibitor.
Leitte et al. [29]	13 November 2019	Cellular culture	One human and two human GBM cell lines from European cellular banks	Human MG cell line, CHME3	Not specified	Results confirm the role of MG in GBM behavior using human cell lines cultured in human serum-based conditions and validate the use of in vitro controllable platforms that recapitulate the microenvironment of GBM as powerful tools for cancer studies.	CHME3 cells were shown to be responsive to classical proinflammatory signals through the Toll-like 4 receptor (TLR4) stimulation by LPS and IFN-γ, leading to activation of STAT1 and the nuclear factor-kb (NF-kb). When challenged with cytotoxic agents, GBM cells showed an increase in proliferation when in contact with MG. Even a low amount of MG (10%–20%) is able to confer resistance of GBM to cytotoxics.
Chiavari et al. [35]	3 November 2020	Cellular culture and histopathology	18	GAMs	Tumor and normal-appearing brain	The data showed the expression of PDIA3 not only in tumor cells but also in GAMs, supporting its potential role in cellular and molecular processes related to GB.	Data showed the expression of PDIA3 not only in tumor cells but also in GAMs, supporting its potential role in cellular and molecular processes related to GB.
Fu et al. [36]	7 May 2020	Mass cytometry	16	GAMs, T cells, NK cells	Tumor and peripheral blood	Recurrent and initial GBMs shared similar immune signatures (the cell types were identified based on the following parameters: T cells, CD45+ CD3+; natural killer (NK) cells, CD45+ CD3-CD16+ CD56+ (10, 19); B cells, CD45+ CD19+; monocytes, CD45+ CD14+ CD16+ (20); macrophages or microglial cells, CD45+ CD11b+ CD3-CD19- CD66b- (15); Tregs, CD45+ CD4+ CD25+ CD127- (21), and granulocytes, CD45+ CD66b+); however, the proportion of GAMs in the recurrent GBMs was decreased compared with that in initial GBMs.	This study confirmed that GAMs, as the dominant infiltrating immune cell population, exhibit substantial inter- and intratumoral heterogeneity in the GBM immune microenvironment, and increased proportions of exhausted T cell subpopulations and Tregs substantially contribute to local immune suppressive characteristics.
Chen et al. [26]	18 November 2020	Cellular culture	2 (GBM12 and GBM39)	Not specified	Not specified	In the study, the combination of increased proliferation but decreased invasion aligns with the go-or-grow hypothesis but more importantly demonstrates that crosstalk between MG and GBM cells in the tumor microenvironment may have powerful effects on GBM activities tied directly to tumor progression and patient survival.	GO analyses revealed GBM-MG co-culture upregulated genes in a patient-derived GBM specimen associated with cell cycle, RNA and DNA division, and metabolic activity. However, genes involved in cell adhesion and migration showed significant downregulation as a result of GBM-MG co-culture.
Tan et al. [37]	21 December 2020	Genomics	9	Macrophages	Not specified	A prognostic model made of six genes was constructed to predict the outcomes of LGG and these are correlated with immune checkpoints which provide a valuable role in diagnosis, prognosis, and immunotherapy of glioma.	The phenotype of macrophages was related to whether the cell is neoplastic or not. The M2 macrophages are mainly gathered in neoplastic cells, while the M1 macrophages are located in non-neoplastic cells. CD163, FPR3, LPAR5, P2ry12, PLAUR, SIGLEC1. CD163, FPR3, SIGLEC1 are correlated to the M2 phenotype
Wei et al. [38]	14 April 2021	Cellular culture and histopathology	63	Macrophages	Cell lines from biobank	This study reports a new mechanism of endothelial cell activation in GBM, which is mediated by TNFα secreting GAMs.	This study shows that GBM cells secrete two important cytokines, IL-8 and CCL2, which stimulate GAMs to produce TNFα. Secreted TNFα then activates ECS to express a gene signature indicative of EC activation, including increased expression of VCAM1, ICAM1, CXCL5, and CXCL10.
C.-k. Shen et al. [17]	28 May 2021	Cellular culture	Not specified	Macrophages	U251 human GBM cells, u87 human GBM cells, thp-1 human monocyte cells	Propose the regulatory mechanism of IL-1β for interaction between GBM and TAM	Il-1β induces the expression of ICAM-1 and VCAM-1 in GBM by binding to the IL-1 receptor, which may play key roles in interacting with GBM and tumor-associated immune cells.
Senjor et al. [39]	29 June 2021	RT-qpcr	Not specified	Macrophages	Not specified	The data show that cystatin F mRNA and protein levels are increased in GBM tissues compared to those in nonmalignant brain tissues. We found that the levels of the transcription factor C/ebpα also increased with disease progression. Cystatin F is expressed by immune cells, differentiated GBM cells, and undifferentiated GSCS	Elevated mRNA levels of Cystatin F and its transcription factor C/ebpα were observed in advanced gliomas compared to nonmalignant brain tissues and in GSCS compared to differentiated GBM cells. Cystatin F was expressed in cells positive for CD68, Iba-1, GFAP, CD44, and SOX-2.
Tanaka et al. [40]	29 June 2021	Qrt-PCR and immunohistochemistry	22 humans	GAMs	15 tumors, seven epileptic surgeries	Peripheral IMG cells obtained by the authors’ previously developed technique can be used to gauge the properties of PMG from the tumor lesion microenvironment in the CNS.	The specific immune status of glioma might be monitored using peripheral IMG cells. There is specific upregulation of CD206 in IMG cells isolated from the peripheral blood of patients with glioma. Synchronous upregulation of CD206 expression levels was observed in most patients with glioma (6/9, 66.7%) and almost all patients with glioblastoma (4/5, 80%).
E. Nuñez et al. [41]	7 December 2021	Cellular culture	20	Macrophages	Not specified	Microglia-derived EGF, PDGFβ, SDF-1α, and IL-6 were identified as the primary activators driving PYK2 and FAK activation in glioma.	Microglia treated with glioma-conditioned medium (GCM) from primary cell lines increased the expression of genes encoding PDGFβ, SDF-1α, IL-6, IL-8, and EGF.
Liu et al. [42]	14 September 2021	Cellular culture and srna sequencing	Five individuals diagnosed with glioma + glioma specimens from 165 chemo- and radiotherapy-naive patients undergoing craniotomy from August 2016 to February 2020 (among these 21 fresh tissues were collected for the following cell culture)	Macrophages and monocyte-derived TAMs	Tumor	Targeting the HGG-AM may represent a promising therapeutic approach for agbm.	HGG-AM was demonstrated to be predominantly enriched in IDH-WT GBM and activated by SETD2-mut tumor cells via TGF-β1 secretion. P2RY12 expression was dramatically compromised in HGG-AM, suggesting a specific microglia activation driven by IDH-WT cancer cells. HGG-AM expressed PDGFRα, suggesting their proliferative capacity.
Urbantat et al. [43]	16 October 2021	Histopathological	76 samples from 38 patients	TAMs	Paired initial and recurrent GBM	A significantly decreased infiltration of tumor-associated microglia and macrophages (TAM, identified through Iba-1) was observed in recurrent tumors, while a high TAM infiltration in primary tumors was associated with a reduced OS.	The analysis of 76 matched primary and recurrent GBM samples underlined important morphological differences between primary and recurrent GBM, with a higher infiltration of TAMs in the primary tumors. Infiltration of TAMs in primary tumors served as a negative predictor for patients’ OS.

**Table 3:** Microglia migratory behavior and its activation pattern.

Author	Year (dd-mm-yyyy)	Type of Study	Sample Size	Microglia Population	Sample	Outcomes	Observations
Parney et al. [44]	Mar 2009	Cellular culture	Nine humans	Macrophages	Tumor only	It has been reported that primary glioma cultures have immunological characteristics that are lost in successive passages.	Monocyte-lineage cells formed the most prominent group of inflammatory cells infiltrating human gliomas. Unexpectedly, it was found that these cells were predominantly CD45bright/CD11b+. This phenotype corresponds to infiltrating systemic macrophages.
X. Yuan et al. [45]	1 December 2016	Histopathological	33	Macrophages	Tumor	From the patient profiles, specific combinations of expression were found, both throughout the tumor sample, and when specifically comparing the adjacent and bulk tissue regions it was possible to predict poorer or better survival.	Higher coverage of astrocytes in adjacent tissue regions increased the hazard of death, whereas positive staining within the tumor had no effect on survival. Increased coverage of microglia adjacent to the tumor decreased the hazard of death while microglia in the tumor increased the hazard of death.
Guo et al. [46]	2 September 2016	Cellular culture	47	Macrophages and monocyte-derived TAMs	Tumor and five normal brain tissues	Demonstrated that hypoxia enhanced the recruitment of TAMs by upregulating POSTN expression in glioma cells. The hypoxic glioma microenvironment polarized TAMs toward the M2 subtype by increasing the expression of M-CSFR in macrophages and TGF-β in glioma cells.	The enhanced directional migration of macrophages toward hypoxic areas has been attributed to the hypoxia-inducible expression of POSTN in glioma cells which may partially explain the mechanism by which macrophages become trapped in hypoxic regions after they were initially attracted to them.
C. Mignogna et al. [47]	23 February 2016	Histopathology and immunohistochemistry	37	Macrophages	Tumor only	Define the distribution and polarization of TAMs in GBM	Immunohistochemical evaluation of macrophage phenotype showed an interesting scenario; the number of cd163+ macrophages was significantly higher (*p* < 0.001) than those of cd68+ macrophages in the perinecrotic area, parenchyma, and, in particular, in perivascular areas. In confocal analysis, the cd163+ population consisted of both cd163+/cd68+ macrophages and single stained cd163+ cells. Interestingly, this finding was more evident in perivascular areas.
L. Lisi et al. [48]	2 March 2017	Hisopathology and immunohistochemistry	41	Macrophages	Tumor core and healthy brain	Results suggest that cd163 expression is higher within the tumor than in the surrounding periphery in both male and female patients.	In the present work, we show that cd163 expression is higher within GBM specimens than in the surrounding periphery in both male and female patients. We report that both inos (m1 marker) and arg-1 (m2 markers) are present both within the tumor and in peripheral parenchyma, albeit unevenly distributed.
Annovazzi et al. [49]	12 September 2017	Histopathological	108	Macrophages	Tumor and 10 healthy controls	Characterization of different patterns of microglia composition based on the degree of infiltration.	The transition from HIA (High infiltration area) to ST (solid tumor) was marked by an almost disappearance of RM (reactive microglia) forms, which could be reduced, destroyed, or switched to serve a different function.
T. Hide et al. [50]	1 March 2018	Genomics	89	Macrophages	Tumor core, border, and periphery	During tumor growth, opcs (oligodendrocyte precursor cells) and macrophages and microglia migrate and proliferate rapidly in the border region, where they secrete growth factors and cytokines, causing GBM cells to acquire stem cell profiles and chemo-radio resistance.	The rapid reaction potential of opcs and macrophages and microglia provides advantages for GBM cells in the formation of the GSC niche in the border because GBM cells possess higher proliferation and migration potential, thereby necessitating rapid adaptation of supportive cells.
Yu-Ju Wu et al. [51]	8 October 2019	Cellular culture	47	Macrophages	Tumor only	Demonstration that CCL5-regulated glioma migration and invasion are associated with the expression levels of p-PYK2 and MMP2.	Newly diagnosed cases with a high CCL5 level were associated with increased tumor volume. GAMs of GBM were involved in the production of CCL. It was observed that high-grade glioma contained a higher level of GAM infiltration. Glioma cells elicited a more robust homing response toward GM-CSF–activated GAM-CM.
Landry et al. [52]	11 November 2020	Genomics	14 tumors	Macrophages	Tumor core and tumor periphery	Analyze geographical differences in macrophage recruitment and activation through sequential activation states. Find distinct activation and maturation processes between tumor core and periphery.	It was found that TAMs in the tumor core mostly originate from the bone marrow-derived pool whereas those in the tumor periphery are largely derived from microglial cells, supporting prior research. Cells in the tumor core evolve from a “pre-activation” state toward a proinflammatory state. Tumor periphery, by contrast, it was found that cells transition from a preactivation state towards pro-oncogenic activation (M2 or “alternative” activation state).
Tang et al. [53]	8 April 2021	Genomics	133	Macrophages	Databases from CCGA	The study comprehensively analyzed the rGBM microenvironment gene signatures, and integrated rGBM microenvironment-associated genes and up-degs in rGBM to identify a novel prognostic immune-related gene, LRRC15.	Confirmed the negative survival role of LRRC15 as an independent prognostic factor, which indicated that LRRC15 could be used as a novel biomarker for predicting the rGBM patients’ outcomes.
K. Kai et al. [54]	30 September 2021	Cellular culture	Not specified	TAMs	Human GBM cell lines t98g and u251 obtained from atcc	Tam-derived il-1β increases growth rate of gbm, promotes TAMs infiltration in the TME and shows higher expression patterns in perinecrotic areas	Involvement of il-1β in GBM (cell lines t98g and u251, with emphasis on u251) growth by means of in vitro studies.

## Abbreviations

APCs	Antigen presenting cells
BBB	Blood brain barrier
CAMs	cell adhesion molecules
CNS	Central nervous system
ECM	Extracellular matrix
EMT	Epithelial–mesenchymal transition
EGFR	epidermal growth factors receptor
FDA	Food and Drug Administration
GAG	Glycosaminoglycans
GAM	Glioma-associated macrophage and microglia
GASC	Glioma Associated Stem cells
GBM	Glioblastoma
GCS	Glioma stem cells
MET	mesenchymal–epithelial transition
PD1	Programmed cell Death-1
PRISMA-P	Preferred Reporting Items for Systematic Review and Meta-Analysis Protocols
SIRT1	Sirtuin 1
TCR	T cell receptors
TGF-β	Transforming growth factor-β
TIL	Tumor-infiltrating lymphocytes
TME	Tumor Microenvironment
TMZ	Temozolomide
